# Point-of-care testing for COVID-19: a simple two-step molecular diagnostic development and validation during the SARS-CoV-2 pandemic

**DOI:** 10.1590/0074-02760230236

**Published:** 2024-10-04

**Authors:** Andre Akira Gonzaga Yoshikawa, Sabrina Fernandes Cardoso, Lívia Budziarek Eslabão, Iara Carolini Pinheiro, Priscila Valverde, Gisele Caminha, Oscar Bruna Romero, Leandro Medeiros, Luísa Damazio Pitaluga Rona, André Nóbrega Pitaluga

**Affiliations:** 1 Universidade Federal de Santa Catarina, Departamento de Biologia Celular, Embriologia e Genética, Florianópolis, SC, Brasil; 2 Secretaria de Saúde do Estado de Santa Catarina, Diretoria de Vigilância Epidemiológica, Florianópolis, SC, Brasil; 3 Universidade Federal de Santa Catarina, Departamento de Microbiologia, Imunologia e Parasitologia, Florianópolis, SC, Brasil; 4 Secretaria Municipal de Saúde, Florianópolis, SC, Brasil; 5 Laboratório Central de Saúde Pública de Santa Catarina, Florianópolis, SC, Brasil; 6 Instituto Federal de Educação, Ciência e Tecnologia de Santa Catarina, Florianópolis, SC, Brasil; 7 Conselho Nacional de Desenvolvimento Científico e Tecnológico, Instituto Nacional de Ciência e Tecnologia em Entomologia Molecular, Rio de Janeiro, RJ, Brasil; 8 Fundação Oswaldo Cruz-Fiocruz, Instituto Oswaldo Cruz, Rio de Janeiro, RJ, Brasil

**Keywords:** COVID-19, SARS-CoV-2, point-of-care molecular diagnostic, RT-LAMP

## Abstract

**BACKGROUND:**

During the coronavirus disease 19 (COVID-19) pandemic, diagnostic testing of the general population proved challenging due to limitations of the gold-standard diagnostic procedure using reverse transcription real-time polymerase chain reaction (RT-qPCR) for large-scale testing on the centralised model, especially in low-resource areas.

**OBJECTIVES:**

To address this, a point-of-care (PoC) diagnostic protocol for COVID-19 was developed, providing fast, reliable, and affordable testing, particularly for low-mid develop areas.

**METHODS:**

The PoC diagnostic process combines a simple paper-based RNA extraction method housed within a 3D-printed plastic device with a colorimetric reverse transcription loop-mediated isothermal amplification (RT-LAMP) assay. Nasopharyngeal/oropharyngeal swabs (NOS) and saliva samples were tested between 2020 and 2021, with the assistance of Santa Catarina’s State Health Secretary, Brazil.

**FINDINGS:**

The developed diagnostic protocol showed a limit of detection of 9,900 copies and an overall diagnostic specificity of 98% for severe acute respiratory syndrome coronavirus 2 (SARS-CoV-2) from 1,348 clinical analysed samples. The diagnostic sensitivity was 95% for NOS samples, 85% for early morning saliva, and 69% for indiscriminate saliva.

**MAIN CONCLUSIONS:**

In conclusion, the developed device successfully extracted SARS-CoV-2 viral RNA from swabs and saliva clinical samples. When combined with colorimetric RT-LAMP, it provides results within 45 min using minimal resources, thus delivering a diagnostic kit protocol that is applicable in large-scale sampling.

In March 2020, the World Health Organization (WHO)^1^ officially announced the global pandemic status of the coronavirus disease 2019 (COVID-19), which is caused by the Betacoronavirus severe acute respiratory syndrome coronavirus 2 (SARS-CoV-2) and transmitted through the air.^2^ In 2021, Latin America, India, and Africa surfaced as the epicentres of this pandemic, wherein there were notable records of reported cases.^3^ In Brazil, the mortality rates associated with COVID-19 were subject to significant concern, primarily due to the increased frequency of death misclassification.^4^ Moreover, it is worth noting that the epidemic’s progression was heterogeneous across different Federal Units.^5^ Furthermore, the overall mortality rates witnessed an upswing, which can be attributed to the indirect COVID-19-related fatalities caused by the overburdening of the healthcare system.^6^

One of the primary challenges in mitigating the pandemic is establishing reliable methods for testing and surveying infected patients and enhancing testing capabilities.^6^ ^,^ ^7^ However, the current gold standard diagnosis procedure, reverse transcription real-time polymerase chain reaction (RT-qPCR), is time-consuming, expensive, and relies on high-cost equipment and qualified laboratory personnel. Additionally, samples often require processing in central laboratories, leading to sampling overload.

During the COVID-19 pandemic, innovative diagnostic alternatives were developed to obtain faster, simpler, and more affordable diagnostic results.^8^ ^,^ ^9^ ^,^ ^10^ In this scenario, a potentially beneficial approach would be to utilise molecular diagnostics in a point-of-care (PoC) mode, with a low-cost, easy, and fast RNA extraction procedure, followed by isothermal amplification of a molecular target (*e.g.*: reverse transcription loop-mediated isothermal amplification (RT-LAMP).^11^ ^,^ ^12^ ^,^ ^13^ Isothermal amplification represents a favourable alternative for PoC given that the equipment involved is more cost-effective, exhibits superior processing speed, and is characterised by heightened ease of use. Also, several studies have demonstrated that the detection of SARS-CoV-2 through RT-LAMP, with a colorimetric readout, yields sensitive and specific outcomes that are comparable to those of reverse transcription-polymerase chain reaction.^3^ ^,^ ^10^ ^,^ ^14^ ^,^ ^15^ ^,^ ^16^ Besides, the saliva utilisation (instead of nasopharyngeal swabs) emerged as an effective solution for PoC devices, since (i) the virus is present and replicates in salivary glands, (ii) it is a non-invasive, and (iii) an easy self-collecting sample.^17^ ^,^ ^18^ ^,^ ^19^

In this study, the development of a PoC molecular testing method for COVID-19 is presented. This approach utilises paper-based RNA extraction, housed on a 3D-printed biodegradable plastic cassette, providing a simple and rapid method for extracting SARS-CoV-2 RNA. After the RNA extraction, a colorimetric RT-LAMP assay targeting three genomic regions of the SARS-CoV-2 genome is conducted. This diagnostic test was developed and validated during the COVID-19 pandemic and has proven to be a simple, robust, and consistent molecular diagnostic test, delivering results from nasopharyngeal swabs or crude saliva within 45 min. This molecular testing kit offers an accessible, easy, and simple PoC option to improve diagnosis in low-resource areas or during high demand for diagnostics.

## SUBJECTS AND METHODS

Samples

*SARS-CoV-2 genome control* - The COVID-19 diagnosis was set up and established by evaluating the sensitivity of the RT-LAMP colour-change diagnostic method, using a synthetic SARS-CoV-2 RNA genome (Twist Bioscience #102024) as RNA positive control. Multiple serial dilutions of the RNA genome control were used to test the entire pipeline.

*Clinical sampling* - All samples used in this study were obtained from suspected cases of COVID-19 or Severe Acute Respiratory Syndrome, collected for official diagnosis purposes by the Central Laboratory of Public Health Authority of Santa Catarina State (Brazil) (LACEN-SC). Samples were collected between the third and eighth day after the onset of symptoms, following the Brazilian Health Authorities’ protocols.^20^

*Clinical blind samples* - To validate the diagnostic protocol with clinical samples, Nasopharyngeal or/and oropharyngeal swab (NOS) blind samples, maintained in Viral Transport Medium (VTM), were kindly donated by LACEN-SC. Those samples were randomly selected from patients who sent samples for official COVID-19 diagnosis throughout Santa Catarina State. Sample processing, RNA extraction, and RT-qPCR assay performed by LACEN-SC followed the Ministry of Health’s recommendation and used either the Charité Institute or the United States Centre of Diseases Control protocols,^20^ with commercial RNA extraction kits. All donated samples were tested in parallel with the diagnostic kit protocol from this study and then compared with the official diagnostic.

*Clinical samples* - To test the fully operational diagnostic kit in field situations, different clinical samples were collected and tested. Biological samples were obtained through a second nasopharyngeal swab (NS) collection from the same patient after the official sample collection was performed and later sent to LACEN-SC. The NS was collected and only the swab tips were transferred to sterile tubes containing 100 μL of lysis buffer (1.75M GuSCN, 66.7% 2-propanol, and 1x RNASecure) and stored at -20ºC until analysis. Indiscriminate saliva (IS) was collected throughout the day with no restriction, and early morning saliva (EMS) collected before feeding, water consumption, or dental hygiene was also evaluated. Saliva samples were self-collected in plastic tubes and sent to the laboratory for analysis. Samples from NS and IS were collected from the same patients at the Tubarão Municipality COVID-19 test facility, and EMS samples were collected from patients at Nossa Senhora da Conceição Hospital (Santa Catarina State, Brazil) with the support of the local health authority (SMS-Tubarão). Additionally, IS and EMS were collected at the Florianópolis Municipality COVID-19 test facility (Santa Catarina State, Brazil) with the support of the local health authority (SMS-Florianópolis).

Ethics approval and consent to participate

All individuals who provided samples for this study were fully informed about the experimental procedures and provided their consent before participation. The development of this project was conducted in compliance with all ethical and personal data protection requirements, which were carefully evaluated by the ethical committee (CAAE: 30764120.2.0000.0121). This ensured that the research adhered to the highest standards of ethics and safety for both human subjects and personal data.

Sample processing and RNA extraction

**Fig. 1:**
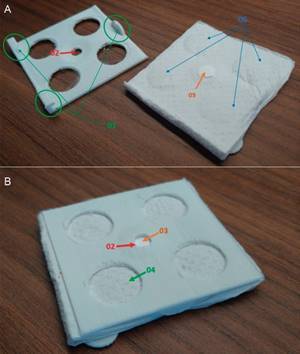
the 3D-printed RNA-extraction cassette. The two distinct parts of the 3D-printed cassette are visualised open in (A), where the hitch parts (01) are observed in the piece on the left, and the pressure exerted on the absorbent pad (06) in the right piece. It is also possible to view the protuberance (05) with the absorbent paper and the polyether sulfone (PES) membrane, and the sample port with a basin around (02). The assembled cassette is visible on (B), to which the sample port (02), the PES membrane (03), and the absorbent paper (04) can be observed.

To validate the PoC pipeline, three RNA extraction methods were employed as follows: (i) Ethanol precipitation with centrifugation was used as a control group since it involves ethanol and glycogen for RNA precipitation similar to the cassette-based extraction method; (ii) an RNA-extraction cassette method using a 3D printed device instead of a centrifuge, aiming to meet PoC requirements; and (iii) a commercial kit - Column extraction was employed to evaluate the sensitivity of the entire pipeline, providing a benchmark for comparison with the RNA-extraction cassette method. Each RNA extraction method is detailed below and the experimental design is explained in Supplementary data (Fig. 1).

*Ethanol precipitation* - The RNA extraction protocol was based on an alcohol precipitation assay adapted from Cao et al.^21^ and Rodriguez et al.^22^ This extraction method was performed on NOS, NS, and IS samples. The swab tips of the NS were placed into sterile tubes containing 100 μL of lysis buffer, effectively inactivating the virus while preserving viral RNA. For the IS samples, 33 μL of saliva was added to a plastic tube (1.5 mL) containing 100 μL of lysis buffer, while for the VTM samples from LACEN-SC, 25 μL of VTM was added to a plastic tube (1.5 mL) containing 75 μL of lysis buffer. Glycogen co-precipitant at 20 mg/mL (Sigma-Aldrich G1767) was added to each mixture and centrifuged at 15,800 g for 15 min. The resulting supernatant was removed, and 100 μL of 70% ethanol was added to the tube and centrifuged at 15,800 g for 5 min. This process was repeated with 100 μL of 100% ethanol, after which the supernatant was removed, and the tubes were left open to air-dry for 10 min at room temperature on the benchtop. Finally, the RNA pellets were resuspended in 50 μL of nuclease-free water. All centrifugation steps were performed at 4ºC using an Eppendorf centrifuge model 5415R.

*RNA-extraction cassette* - To assemble a PoC diagnostic kit, a 3D-printed plastic device for paper-based RNA extraction was developed to carry out the RNA extraction assay (Fig. 1 - .stl archive available upon request). Fig. 1A shows the hitch parts (01) in the piece on the left, as well as the pressure applied by the absorbent pad (06) on the piece on the right. Furthermore, the left piece contains a protuberance (05) with both an absorbent paper (any paper towel), and a Polyether Sulfone (PES) membrane (MERK, GPWP14250), as well as a sample port with a basin around it (02). In Fig. 1B, the assembled device is displayed, including the sample port (02), the PES membrane (03), and the absorbent paper (04). This depiction provides a clear visual representation of the various components of the cassette and its positioning. The circular PES filter paper (Fig. 1) with a diameter of 7 mm and a pore size of 0.22 μm was placed on a 6 cm^2^ absorbent paper pad to retain approximately 400 μL of liquid waste. The plastic device used for RNA extraction was designed and 3D printed on a GTMAX3D CORE A2V2 printer using the biodegradable and plant based polylactic acid polymer (PLA) (Fig. 1). It has a surface area of 6.1 cm^2^ split into two layers: an upper part with a circular sample entrance of 6 mm diameter for the PES filter paper, and a lower part designed to house the PES membrane and the absorbent pad.^22^ The designed cassette has been patented pending (BR 10 2021 00889), entitled “Molecular diagnostic point-of-care testing kit for Covid-19,” by the World Intellectual Property Organization (WIPO).^23^

The RNA-extraction cassette procedure employed an adapted ethanol precipitation protocol. The lysis buffer was mixed with either saliva or VTM samples in a 1:3 ratio: 25 μL of VTM to 75 μL of buffer, and 33 μL of saliva to 100 μL of buffer. Glycogen co-precipitant at 20 mg/mL (Sigma-Aldrich G1767) was added to each mixture, which was then pipetted into the entrance port of the PES membrane. Capillary action drew the liquid phase through the membrane, while the solid phase composed of the RNA-Glycogen precipitate was retained on the PES membrane. The PES membrane was subsequently washed with 150 μL of 70% ethanol and 100 μL of 100% ethanol sequentially. The absorbent pad absorbed the liquid phase during each rinse. Thereafter, the PES membrane was transferred to a 1.5 mL plastic tube. The tubes were left open for 1 minute to allow the PES membrane to air-dry. Finally, 50 µL of nuclease-free water was added to the tube and resuspended for 30 s to release the RNA from the PES membrane.

*Commercial kit - Column extraction* - The efficiency of the developed RNA-extraction cassette was evaluated by comparing its results with those using the Qiagen RNeasy Mini Kit (QIA74104), following the manufacturer’s instructions. All viral RNA standardised control samples used in this study were SARS-CoV-2 full nonoverlapping genome fragments (Twist Bioscience #SKU 102019), with 1 × 10^6^ RNA copies per microliter, diluted appropriately in nuclease-free water. Triplicate RNA extraction assays using different sample concentrations of SARS-CoV-2 control RNA (100, 500, 1,000, 5,000, 25,000, and 50,000 copies) were simultaneously performed using both the Qiagen RNeasy Mini Kit and RNA-extraction cassette. Then, the samples were submitted to the optimised Colorimetric RT-LAMP assay to evaluate the limit of detection (LoD) of the RNA extraction procedure. To further evaluate the analytical sensitivity of the RNA-extraction cassette, the experiment was repeated using saliva from a healthy patient. It was tested in five replicates with sample concentrations of 3,300, 6,600, 9,900, and 13,200 viral RNA copies.

Colorimetric RT-LAMP

The COVID-19 specific primers (As1e, N2, and E1) targeting the viral genome positions *Orf1a*, *N*, and *E*, respectively, and described by Rabe & Cepko^24^ and Zhang et al.^25^ were used. All primers were resuspended in nuclease-free water and combined to make a 10x primer mix as follows for each set: FIP and BIP (16 μM each), F3 and B3 (2 μM each), LF and LB (4 μM each). The LAMP assay was carried out in a final volume of 20 μL containing: 2 μL from each of the three 10x primer mixes, 10 μL of WarmStart Colorimetric Lamp 2X Master Mix (M1800), and 4 μL of the target RNA. The reaction was carried out at 65ºC for 30 min in a dry bath (Kasvi). Every RT-LAMP assay was executed with a negative control (replacing RNA with nuclease-free water), and a positive control (1,000 RNA copies per reaction of the Synthetic SARS-Cov-2, Twist Bioscience #SKU 102019). The colorimetric RT-LAMP assay allows a naked-eye analysis of the results with pink as negative and yellow as positive samples. Different colours, such as orange, were considered inconclusive. To avoid misinterpretation, the same group of people were responsible for all colorimetric results analysis.

Validation of the LAMP assay with clinical samples

To validate the diagnostic process, a test was conducted on 1,348 clinical samples classified into six categories based on the type of sample, origin, and RNA extraction method, as follows: (1) nasopharyngeal swab samples collected in Tubarão and extracted by ethanol precipitation using centrifugation (NSC); (2) indiscriminate saliva samples collected in Tubarão and extracted by ethanol precipitation using centrifugation (ISC); (3) nasopharyngeal and oropharyngeal swab samples provided by LACEN-SC and extracted by ethanol precipitation using centrifugation (NOSC); (4) nasopharyngeal and oropharyngeal swab samples provided by LACEN-SC and extracted with RNA-extraction cassette (NOSD); (5) indiscriminate saliva samples collected in Florianópolis and extracted with RNA-extraction cassette (ISD) and (6) early morning saliva collected in Florianópolis and extracted with RNA-extraction cassette (EMSD) [Supplementary data (Fig. 1)].

Statistical analysis

To evaluate the accuracy of the RT-LAMP assay, the results obtained were compared with those of the RT-qPCR assay performed by LACEN-SC. The comparison was done for true positive (patients positive in both RT-LAMP and RT-qPCR), true negative (patients negative in both RT-LAMP and RT-qPCR), false positive (patients negative in RT-qPCR but positive in RT-LAMP), and false negative (patients positive in RT-qPCR but negative in RT-LAMP) cases, following the methodology described by Lakens.^26^ The diagnostic sensitivity, specificity, negative predictive value, and positive predictive value were calculated using MedCalc Statistical Software version 19.2.6 (MedCalc Software bv, Ostend, Belgium; www.medcalc.org; 2020). Additionally, Cohen Kappa was calculated using the VassarStats platform (available at http://vassarstats.net/).

## RESULTS

COVID-19 PoC diagnostic kit LoD

**Fig. 2:**
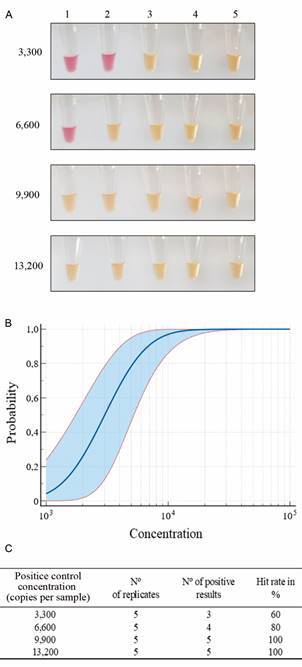
limit of detection (LoD) of the RNA-extraction cassette method using control RNA and saliva from a healthy patient. (A) Reverse transcription loop-mediated isothermal amplification (RT-LAMP) colorimetric results from control RNA dilutions extracted with the RNA-extraction cassette. (B) Probit regression analysis curve considering five independent replicates with different copies per sample. (C) Table with the hit rate (%) reached in all replicates.

The sensitivity of the COVID-19 PoC diagnostic kit was assessed by comparing the RT-LAMP LoD using the RNA-extraction cassette method with a gold standard commercial kit - Column extraction method (Qiagen RNeasy Mini Kit). This comparison was conducted across various dilutions of the synthetic SARS-CoV-2 genome in nuclease-free water. Following RNA extractions, the colorimetric results of the RT-LAMP assay were analysed [Supplementary data (Fig. 2A)], revealing a LoD of 1,000 copies for the commercial kit - Column extraction method, and 25,000 copies for the RNA-extraction cassette method. However, the *probit* regression analysis suggested that the LoD for the RNA-extraction cassette method would be 9,883.56 (p < 0,0001) copies [Supplementary data (Fig. 2B)]. So, to further establish the LoD of the PoC COVID-19 diagnostic kit, a second experiment was executed in five replicates of positive control in the same fashion as the previous experiment using just the RNA-extraction cassette and saliva from a healthy patient instead of nuclease-free water. The colorimetric RT-LAMP results (Fig. 2A) indicated a LoD of 9,900 copies (or 300 copies/μL), while the *probit* regression analysis resulted in a LoD (within 95% reliability) of 8,633.85 copies (p = 0.0366) (Fig. 2B-C).

Colorimetric RT-LAMP detection of SARS-CoV-2 in clinical samples

A comparative analysis was conducted between the colorimetric RT-LAMP results from this study and the LACEN-SC RT-qPCR results to determine the diagnostic accuracy of the assay. The diagnostic accuracy evaluation involved the calculation of sensitivity, specificity, positive predictive value (PPV), negative predictive value (NPV), and Cohen’s Kappa (Table). In all six sample categories, the specificity was above 97%, and 3% were deemed inconclusive due to orange colour output. The RT-LAMP assay time was optimised to 30 min using the combination of all three primer sets (As1e, N2, and E1),^24^ ^,^ ^25^ compared to the 50 min required when each set of primers was evaluated individually, or 40-45 min when the primers were evaluated in pairs [Supplementary data (Fig. 3)]. So, the time from sample RNA extraction to assay visual result was 45 min using the PoC RNA-extraction cassette method.

The diagnostic results for the RNA extraction methods (i) ethanol precipitation, and (ii) RNA-extraction cassette, are detailed below.

**TABLE:** Reverse transcription loop-mediated isothermal amplification (RT-LAMP) results in parameters

Sample group	NSC	ISC	NOSC	NOSD	ISD	EMSD
S	80.36% (67.57 - 89.77)	70.93% (60.14 - 80.22)	92.59% (85.93 - 96.75)	95.83% (85.75 - 99.49)	69.95% (62.74 - 76.49)	85.71% (42.12 - 99.63)
Sp	97.46% (92.75 - 99.47)	99.44% (96.94 - 99.99)	99.46% (97.01 - 99.99)	99.66% (98.14 - 99.99)	99.66% (98.14 - 99.99)	100% (88.43 - 100)
PPV	93.75% (82.97 - 97.88)	98.39% (89.58 - 99.77)	99.01% (92.87 - 99.85)	97.87% (86.84 - 99.69)	99.22% (94.75 - 99.89)	100%
NPV	91.27% (86.01 - 94.67)	87.75% (83.73 - 90.88)	95.81% (92.15 - 97.81)	96.15% (86.55 - 98.98)	84.33% (81.18 - 87.04)	96.77% (77.60 - 99.14)
CK	0.80 (0.71 - 0.90)	0.75 (0.67 - 0.84)	0.92 (0.88 - 0.97)	0.93 (0.87 - 1)	0.73 (0.67 - 0.80)	0.86 (0.60 - 1)
n	174	266	292	99	480	37
IR	5.9%	6%	0	0	3%	0

NSC: nasopharyngeal swab extracted using ethanol precipitation with centrifugation; ISC: indiscriminate saliva extracted using ethanol precipitation with centrifugation; NOSC: nasopharyngeal and oropharyngeal swab extracted using ethanol precipitation with centrifugation; NOSD: nasopharyngeal and oropharyngeal swab extracted using the RNA-extraction cassette; ISD: indiscriminate saliva extracted using the RNA-extraction cassette; EMSD: early morning saliva extracted using the RNA-extraction cassette; S: sensitivity; Sp: specificity; PPV: positive predictive value; NPV: negative predictive value; CK: Cohen’s Kappa; n: number of samples; IR: inconclusive rate. Values presented in parentheses represent the 95% confidence interval (CI).

*Diagnostic results for RNA extraction by ethanol precipitation in centrifuge* - In this study, the NSC, ISC, and NOSC clinical samples had their RNA extracted using a standard ethanol precipitation method with centrifugation. Also, to reduce patient discomfort and accelerate the collection process, saliva was self-collected by patients in 50 mL plastic tubes indiscriminately, and its viability for COVID-19 detection was tested. The efficiency of saliva as a biological sample for COVID-19 diagnosis was compared to swab samples. A total of 174 swab samples (NSC) and 266 indiscriminate saliva samples (ISC) were analysed to evaluate the performance of the colorimetric RT-LAMP assay for COVID-19 diagnosis. The diagnostic results for NSC samples showed a sensitivity of 80%, specificity of 97%, and Cohen’s Kappa of 0.8, while the diagnostic results for ISC samples showed a sensitivity of 70%, specificity of 99%, and Cohen’s Kappa of 0.75. In addition, a donation of 292 swab blind samples from LACEN-SC (NOSC) collected according to State protocols was received, which were also extracted using ethanol precipitation in a centrifuge. The diagnostic results showed a sensitivity of 92%, a specificity of 99%, and a Cohen’s Kappa of 0.92.

*Diagnostic results for RNA-extraction cassette* - To validate the RNA-extraction cassette method, an analysis of 99 swab samples (NOSD) from LACEN-SC, and 480 saliva samples from SMS-Florianópolis (ISD) was conducted. The combination of the RNA-extraction cassette with the colorimetric RT-LAMP reaction resulted in the development of a portable PoC diagnostic kit. In the NOSD group, the diagnostic kit yielded 95% sensitivity, 99% specificity, and Cohen’s Kappa of 0.93. Saliva samples were tested with the RNA-extraction cassette method to further explore the viability of alternative biological samples. The ISD samples were collected in the same manner as mentioned before, showing 69% sensitivity, 99% specificity, and Cohen’s Kappa of 0.73. To make the saliva samples more standardised and favour the sample’s viral load, early morning saliva samples were collected from suspected COVID-19 cases without food and water consumption or buccal hygiene.^18^ A total of 37 samples (EMSD) were gathered, achieving 85% diagnostic sensitivity, 100% diagnostic specificity, and Cohen’s Kappa of 0.86.

## DISCUSSION

In this study, the aim was to address the challenges posed by the rapid increase in demand for reliable diagnostics to combat the COVID-19 pandemic. A PoC molecular diagnostic kit was developed to facilitate, accelerate, and reduce the cost of SARS-CoV-2 detection, consisting of an RNA-extraction cassette and RT-LAMP assay. Different clinical samples were used to validate a colorimetric RT-LAMP reaction, compatible with a 3D-printed RNA-extraction cassette. Thus, a reliable, simple, and affordable COVID-19 PoC diagnostic kit was developed, enabling the testing of various biological samples.

LoD

The RNA-extraction cassette and RT-LAMP reaction were optimised, achieving a LoD of 9,900 RNA copies (3 × 10^5^ copies/mL or 3 × 10^2^ copies/μL) compared to 1,000 RNA copies when combining a commercial kit - Column extraction method with the RT-LAMP reaction protocol [Fig. 2, Supplementary data (Fig. 2)]. Despite the difference, the LoD of the COVID-19 PoC diagnostic kit consistently detected sufficient RNA for accurate diagnostics, since the achieved LoD was under the observed RNA copy number for fatal cases (3.57 × 10^9^ copies/mL), survived cases (3.92 × 10^8^ copies/μL), and asymptomatic cases (4.92 × 10^7^ copies/μL) of COVID-19 reported by Tsukagoshi et al.^27^ with NS samples (n = 370). Rodriguez et al.^22^ used a similar methodology for RNA extraction for the H1N1 virus and reported a LoD of 10^6^ copies/mL, which falls into the range of the PoC diagnostic kit reported LoD (3 × 10^5^ copies/mL). Furthermore, the LoD of the PoC diagnostic kit falls within the range of COVID-19 saliva samples, reported from 10^4^ up to 10^8^ copies/μL,^28^ indicating that the established LoD can successfully detect COVID-19 positive patients both with NS and saliva samples.

The sensitivity of swab and saliva samples

*Swab samples* - The 1,348 clinical samples analysed were divided into six groups (Table) and were collected in parallel with LACEN-SC sampling, enabling the comparison of the RT-LAMP assay results with the official state health authorities’ RT-qPCR results. The performance of the molecular diagnostic kit was evaluated by focusing on three swab clinical groups: two of them were extracted by ethanol precipitation with centrifugation (NOSC and NSC), and the third was processed using the RNA-extraction cassette (NOSD) (Table). The diagnostic sensitivities achieved were 80% (NSC), 92% (NOSC), and 95% (NOSD), which are consistent with previous studies conducted by Aoki et al.^29^ (79%; n = 62), Dao Thi et al.^10^ (72%; n = 768), and Chow et al.^15^ (95%; n = 223) using commercial RNA extraction kits and detection through RT-LAMP. The lower sensitivity of 80% detected in Tubarão swab samples (NSC) may be due to their second examination from the same patient using a thinner swab. It is possible that the second collection with a smaller swab resulted in a reduced viral load. Previous work by Dao Thi et al.^10^ showed that a colorimetric RT-LAMP protocol with NS achieved a sensitivity of 97% in samples with Ct values below 30. However, the overall sensitivity was lower, likely due to higher Ct values, which represent lower viral loads and thus lower diagnostic sensitivity. Overall, the high sensitivity obtained from LACEN-SC VTM samples (NOSC and NOSD: 92% and 95%, respectively) demonstrates that the ethanol precipitation and RNA-extraction cassette methods were successful and efficient, especially when used for the first examination.

*Saliva samples* - The viability of saliva as an alternative specimen for COVID-19 detection was also evaluated. Three of the six clinical groups analysed in this study (Table) comprised saliva samples: two of them involved IS, and one was composed of EMS. The IS samples were submitted to ethanol precipitation with centrifugation (ISC), and using the RNA-extraction cassette method (ISD), resulting in a sensitivity of 70% and 69%, respectively. These results are consistent with the findings of Nagura-Ikeda et al.^30^ who reported similar percentages (70%) for the sensitivity of saliva samples extracted using a commercial kit and tested through RT-LAMP. Another study conducted by Rao et al.^18^ demonstrated that saliva samples collected in the early morning had a higher detection rate in identifying the virus using RT-qPCR when compared to nasopharyngeal swabs. So, to further explore the use of saliva in PoC diagnostic, 37 EMSD samples were collected and processed using the RNA-extraction cassette, resulting in a sensitivity of 85%. This sensitivity aligns with similar findings reported by Santos et al.^19^ (80% for n = 485), and Lalli et al.^31^ (85% for n = 30), both using indiscriminate saliva through RT-LAMP testing. The former study used a commercial RNA extraction kit, while the latter employed an in-house saliva pre-treatment.

Other RT-LAMP parameters

The findings of this study show that both ethanol precipitation with centrifugation and RNA-extraction cassette methods were effective in detecting COVID-19 in swab and saliva samples when combined with the colorimetric RT-LAMP assay.

The PPV values were > 94% for all six groups, which means a < 6% chance of producing a false-positive result (incorrectly identifying a negative case as positive). For NS, NOS, and EMS samples the NPV values were > 91%, resulting in < 9% chance of generating a false-negative result (incorrectly identifying a positive case as negative) (Table).^32^ Analysing the RNA-extraction cassette samples from NOS and EMS, the PPV and NPV values were NOSD = 97% and 96%, and EMSD = 100% and 97%. This means that when combined with NOS in VTM samples or with EMS, the diagnostic kit (from RNA-extraction cassette to RT-LAMP) had a < 3% chance of producing a false-positive result, and a < 4% chance of generating a false-negative result. These results are consistent with Nawattanapaiboon et al.,^16^ who analysed 2,120 NOS COVID-19 samples (nasopharyngeal and throat swabs) using a commercial extraction kit and RT-LAMP and yielded a PPV and an NPV of 99%.

The specificity values for all six groups analysed in this study were > 97.5%, which is consistent with previous COVID-19 research using swabs and saliva samples (specificity: 81% - 100%).^3^ ^,^ ^15^ ^,^ ^31^ It is worth noting that the high specificity values obtained in this study for all six groups may be attributed to the use of three primer sets,^24^ ^,^ ^25^ which are known to decrease nonspecific amplification, thus minimising the risk of false-positive results.

In this study, Cohen’s Kappa coefficient was employed to assess the agreement between two observers in their interpretations of RT-LAMP and RT-qPCR results for positive and negative samples. Cohen’s Kappa coefficient is a widely used measure of reliability.^33^ The results for NOSC (k = 0.92) and NOSD (k = 0.93), as well as EMSD (k = 0.86), were consistent with previous studies of COVID-19 detection using saliva samples conducted by Santos et al.^19^ (k = 0.89) and Kobayashi et al.^34^ (k = 0.88), and nasopharyngeal swab samples conducted by Silva et al.^35^ (k = 0.89). These findings suggest that the RT-LAMP method utilised in this study is highly reliable for COVID-19 detection.

Inconclusive RT-LAMP results

The colorimetric result is a useful approach for naked-eye analysis, where in this study a pink colour indicates a negative result, while a yellow colour indicates a positive result [Supplementary data (Fig. 4)]. However, it should be noted that a third colour, orange [Supplementary data (Fig. 4B)], has been observed in approximately 3% of the results. To address this issue, Cui et al.^9^ have suggested that orange results should be classified as positive, albeit with low viral loads. Meanwhile, Chow et al.^15^ have established that pink or coral pink colour is indicative of a negative result, while yellow or amber colours are indicative of positive results. However, the results were classified according to the Aoki et al.^21^ classification as inconclusive, suggesting that the samples should be reanalysed to prevent mislabelling of results as false positives or false negatives.

In conclusion

The streamlined solution offered by the PoC molecular diagnostic kit combines a user-friendly RNA-extraction cassette with colorimetric RT-LAMP [Supplementary data (Fig. 5)], facilitating decentralised on-site testing at local health centres. Results are provided within an hour using a common dry bath. Originally designed for COVID-19, this adaptable approach is suitable for other viral diseases, especially in low to middle-income countries facing frequent outbreaks. The development, testing, and field use of this PoC tool involved various biological samples and eliminated the need for expensive and time-consuming commercial extraction kits. The efficacy of the diagnostic kit was validated through direct testing with a range of clinical samples, demonstrating its accuracy and affordability for viral diagnosis, particularly in resource-limited areas.

## References

[1] WHO (2020). Director-General's opening remarks at the media briefing on COVID-19. https://www.who.int/director-general/speeches/detail/who-director-general-s-opening-remarks-at-the-media-briefing-on-covid-19---11-march-2020..

[2] Zhu N, Zhang D, Wang W, Li X, Yang B, Song J (2020). A novel coronavirus from patients with pneumonia in China, 2019. N Engl J Med.

[3] González-González E, Lara-Mayorga IM, Rodríguez-Sánchez IP, Zhang YS, Martínez-Chapa SO, Santiago GT (2021). Colorimetric loop-mediated isothermal amplification (LAMP) for cost-effective and quantitative detection of SARS-CoV-2 the change in color in LAMP-based assays quantitatively correlates with viral copy number. Anal Methods.

[4] Orellana JDY, da Cunha GM, Marrero L, Moreira RI, da Costa Leite I.Horta BL (2021). Excess deaths during the COVID-19 pandemic underreporting and regional inequalities in Brazil. Cad Saude Publica.

[5] Lobo AP, Cardoso-Dos-Santos AC, Rocha MS, Pinheiro RS, Breem JM, Macário EM (2020). COVID-19 epidemic in Brazil Where are we at?. Int J Infect Dis.

[6] Barreto ML, Barros AJD, Carvalho MS, Codeço CT, Hallal PRC, Medronho RA (2020). O que é urgente e necessário para subsidiar as políticas de enfrentamento da pandemia de COVID-19 no Brasil [What is urgent and necessary to inform policies to deal with the COVID-19 pandemic in Brazil?]. Rev Bras. Epidemiol.

[7] Carvalho TA, Boschiero MN, Marson FAL (2021). COVID-19 in Brazil 150,000 deaths and the Brazilian underreporting. Diagn Microbiol Infect Dis.

[8] de Oliveira KG, Estrela PFN, Mendes GM, Dos Santos CA, Silveira-Lacerda EP, Duarte GRM (2021). Rapid molecular diagnostics of COVID-19 by RT-LAMP in a centrifugal polystyrene-toner based microdevice with end-point visual detection. Analyst.

[9] Cui Z, Chang H, Wang H, Lim B, Hsu CC, Yu Y (2020). Development of a rapid test kit for SARS-CoV-2 an example of product design. Biodes Manuf.

[10] Dao Thi VL, Herbst K, Boerner K, Meurer M, Kremer LPM, Kirrmaier D (2020). A colorimetric RT-LAMP assay and LAMP-sequencing for detecting SARS-CoV-2 RNA in clinical samples. Sci Transl Med.

[11] Notomi T, Okayama H, Masubuchi H, Yonekawa T, Watanabe K, Amino N (2000). Loop-mediated isothermal amplification of DNA. Nucleic Acids Res.

[12] Nagamine K, Hase T, Notomi T (2002). Accelerated reaction by loop-mediated isothermal amplification using loop primers. Mol Cell Probes.

[13] Tomita N, Mori Y, Kanda H, Notomi T (2008). Loop-mediated isothermal amplification (LAMP) of gene sequences and simple visual detection of products. Nat Protoc.

[14] Huang WE, Lim B, Hsu CC, Xiong D, Wu E, Yu Y (2020). RT-LAMP for rapid diagnosis of coronavirus SARS-CoV-2. Microb Biotechnol.

[15] Chow FW, Chan TT, Tam AR, Zhao S, Yao W, Fung J (2020). A rapid, simple, inexpensive, and mobile colorimetric assay COVID-19-LAMP for mass on-site screening of COVID-19. Int J Mol Sci.

[16] Nawattanapaiboon K, Pasomsub E, Prombun P, Wongbunmak A, Jenjitwanich A, Mahasupachai P (2021). Colorimetric reverse transcription loop-mediated isothermal amplification (RT-LAMP) as a visual diagnostic platform for the detection of the emerging coronavirus SARS-CoV-2. Analyst.

[17] Matuck BF, Dolhnikoff M, Duarte-Neto AN, Maia G, Gomes SC, Sendyk DI (2021). Salivary glands are a target for SARS-CoV-2 a source for saliva contamination. J Pathol.

[18] Rao M, Rashid FA, Sabri FSAH, Jamil NN, Zain R, Hashim R (2021). Comparing Nasopharyngeal swab and early morning saliva for the identification of severe acute respiratory syndrome coronavirus 2 (SARS-CoV-2). Clin Infect Dis.

[19] Santos CAD, Oliveira KGD, Mendes GM, Silva LC, Souza MND, Estrela PFN (2021). Detection of SARS-CoV-2 in saliva by RT-LAMP during a screening of workers in Brazil, including pre-symptomatic carriers. J Braz Chem Soc.

[20] GOV-SC, SES, SUS, SVS, DVE (2022). Manual de orientações da COVID-19 (vírus SARS-Cov-2). https://www.cosemssc.org.br/wp-content/uploads/2022/02/MANUAL-DE-ORIENTAES-DA-COVID-19-2022.pdf..

[21] Cao Q, Mahalanabis M, Chang J, Carey B, Hsieh C, Stanley A (2012). Microfluidic chip for molecular amplification of influenza a RNA in human respiratory specimens. PLoS One.

[22] Rodriguez NM, Linnes JC, Fan A, Ellenson CK, Pollock NR, Klapperich CM (2015). Paper-based RNA extraction, in situ isothermal amplification, and lateral flow detection for low-cost, rapid diagnosis of influenza A (H1N1) from clinical specimens. Anal Chem.

[23] Pitaluga AN, Rona LD, Cardoso SF, Toledo C, Sebastiao LM (2022). Molecular diagnostic point-of-care testing kit for Covid-19. Brazil Patent.

[24] Rabe BA, Cepko C (2020). SARS-CoV-2 detection using isothermal amplification and a rapid, inexpensive protocol for sample inactivation and purification. Proc Natl Acad Sci USA.

[25] Zhang Y, Ren G, Buss J, Barry AJ, Patton GC, Tanner NA (2020). Enhancing colorimetric loop-mediated isothermal amplification speed and sensitivity with guanidine chloride. Biotechniques.

[26] Lakens D, Motulsky H (2018). Intuitive biostatistics: a nonmathematical guide to statistical thinking.

[27] Tsukagoshi H, Shinoda D, Saito M, Okayama K, Sada M, Kimura H (2021). Relationships between viral load and the clinical course of COVID-19. Viruses.

[28] Zhu J, Guo J, Xu Y, Chen X (2020). Viral dynamics of SARS-CoV-2 in saliva from infected patients. J Infection.

[29] Aoki MN, de Oliveira Coelho B.Góes LGB.Minoprio P.Durigon EL.Morello LG (2021). Colorimetric RT-LAMP SARS-CoV-2 diagnostic sensitivity relies on color interpretation and viral load. Sci Rep.

[30] Nagura-Ikeda M, Imai K, Tabata S, Miyoshi K, Murahara N, Mizuno T (2020). Clinical evaluation of self-collected saliva by quantitative reverse transcription-PCR (RT-qPCR), direct RT-qPCR, reverse transcription-loop-mediated isothermal amplification, and a rapid antigen test to diagnose COVID-19. J Clin Microbiol.

[31] Lalli MA, Langmade JS, Chen X, Fronick CC, Sawyer CS, Burcea LC (2021). Rapid and extraction-free detection of SARS-CoV-2 from saliva by colorimetric reverse-transcription loop-mediated isothermal amplification. Clin Chem.

[32] Uribe-Alvarez C, Lam Q, Baldwin DA, Chernoff J (2021). Low saliva pH can yield false positives results in simple RT-LAMP-based SARS-CoV-2 diagnostic tests. PLoS One.

[33] McHugh ML (2012). Interrater reliability the kappa statistic. Biochem Med (Zagreb).

[34] Kobayashi GS, Brito LA, Moreira DP, Suzuki AM, Hsia GSP, Pimentel LF (2021). A novel saliva RT-LAMP workflow for rapid identification of COVID-19 cases and restraining viral spread. Diagnostics (Basel).

[35] Silva LDC, dos Santos CA, Mendes GDM, Oliveira KGD, de Souza Jr MN.Estrela PFN (2021). Can a field molecular diagnosis be accurate A performance evaluation of colorimetric RT-LAMP for the detection of SARS-CoV-2 in a hospital setting. Anal Methods.

