# Supplementary material for: Point-of-care testing for COVID-19: a simple two-step molecular diagnostic development and validation during the SARS-CoV-2 pandemic
Source: Mem Inst Oswaldo Cruz. 2024 Oct 4;119:e230236. doi: 10.1590/0074-02760230236 (PMC11452069; doi:10.1590/0074-02760230236)
Supplement: Supplementary file 1 [file 1678-8060-mioc-119-e230236-s.pdf]

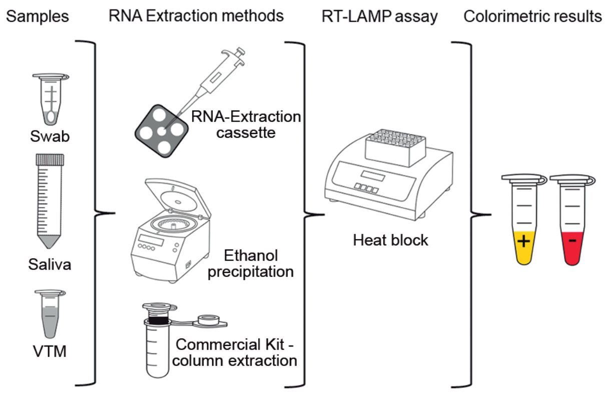

Fig. 1: experimental design. The study included diverse biological samples: nasopharyngeal swabs (NS), indiscriminate (IS) and early morning saliva (EMS), and VTM from nasopharyngeal/oropharyngeal swab collections (NOS). The biological samples were tested with an RNA-extraction cassette method using a 3D printed cassette instead of a centrifuge, aiming to meet point-of-care (PoC) requirements, and an ethanol precipitation with centrifugation used as a control group. The commercial kit - Column extraction was employed with different dilutions of the synthetic RNA in nuclease-free water / healthy saliva to evaluate the sensitivity of the entire pipeline, providing a benchmark for comparison with the RNA- extraction cassette method. The reverse transcription loop-mediated isothermal amplification (RT-LAMP) assay was performed in heating blocks at 65ºC for 30 min using the WarmStart Colorimetric LAMP from NEB (M1800), yielding visual results by phenol red colorimetry: pink as negative and yellow as positive samples.

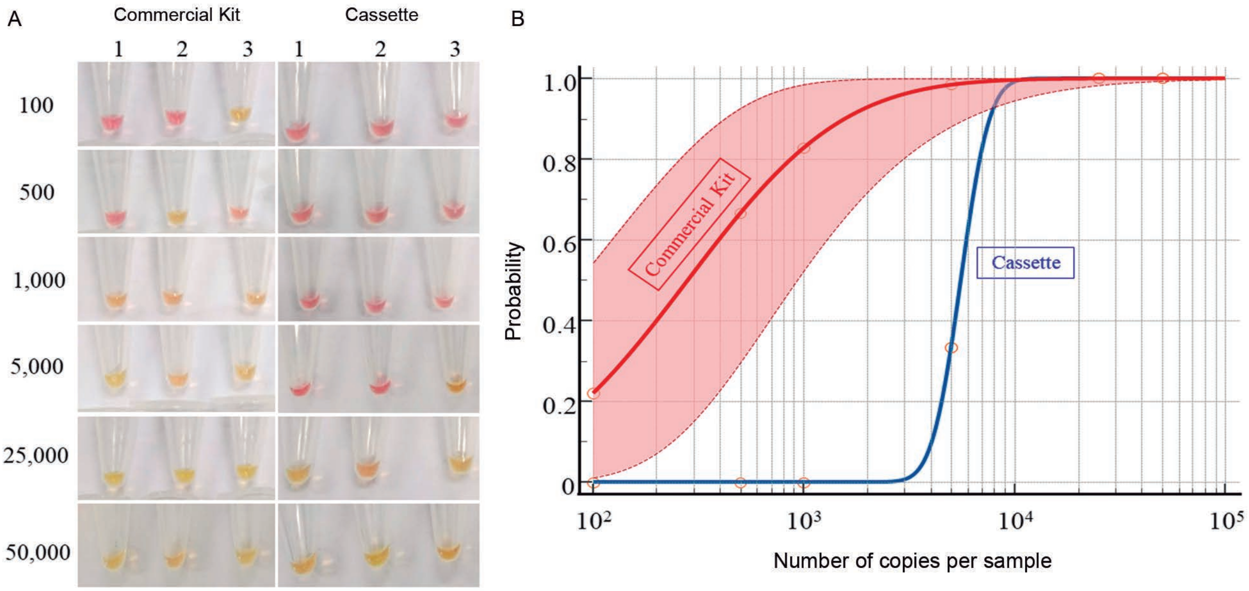

Fig. 2: limit of detection (LoD) of extraction methods using control RNA spiked in nuclease-free water. (A) Reverse transcription loop-mediated isothermal amplification (RT-LAMP) colorimetric results from control RNA dilutions extracted using a commercial kit - Column extraction and the RNA-extraction cassette. (B) Probit regression analysis curve considering three independent replicates with different copies per sample of the commercial kit and the cassette extracted replicates.

CC BY

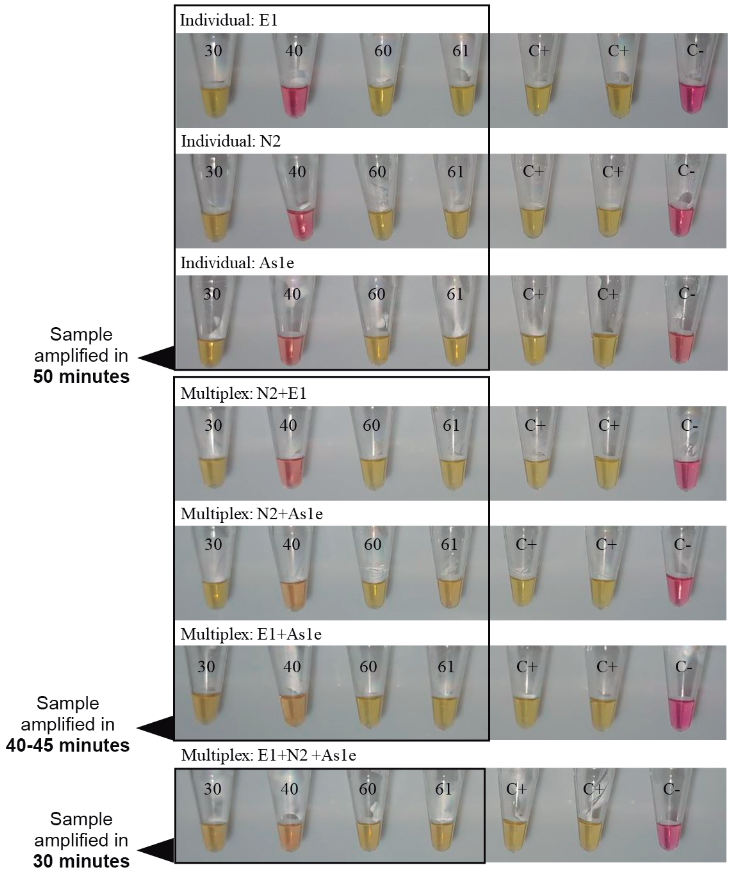

Fig. 3: primers performance test. The efficiency of each primer set were tested individually (E1, N2, As1e, targeting the viral genome positions E, N, and Orf1a, respectively), in pairs (E1+N2, E1+As1e, As1e+N2), and in combination (E1+N2+As1e). Positive results were achieved within 30 min only when using the multiplex assay with the three primer sets. The N2+As1e and E1+As1e combinations yielded positive results within 40-45 min. However, the N2+E1 combination failed to amplify one sample within the same time frame. When tested individually, the primers produced positive results for three of the four samples within 50 min. These findings indicate that the combination of all three primer sets is the most effective method for detecting coronavirus disease 19 (COVID-19) in clinical samples, reducing the time required for diagnosis. 30, 40, 60, 61: four early morning saliva positive samples. C+: two positive controls using synthetic severe acute respiratory syndrome coronavirus 2 (SARS-CoV-2) RNA. C-: negative control with nuclease-free water.

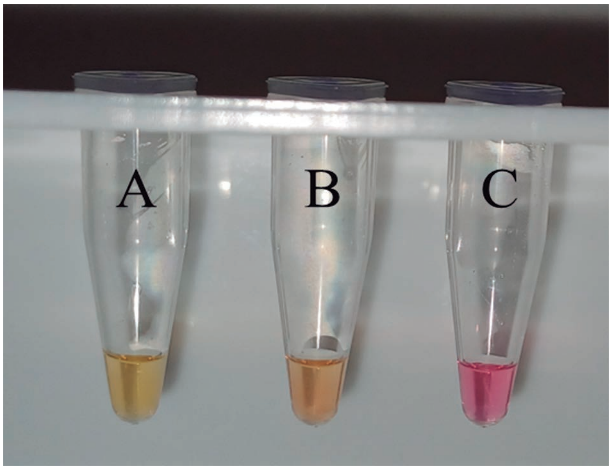

Fig. 4: reverse transcription loop-mediated isothermal amplification (RT-LAMP) colorimetric results. (A) Yellow colour indicating positive results; (B) Orange colour indicating inconclusive results according to Aoki et al.;[21] (C) Pink colour indicating negative results.

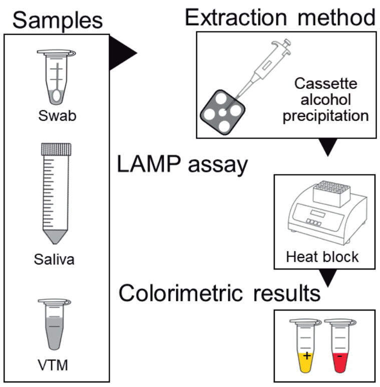

Fig. 5: graphical abstract of the coronavirus disease 19 (COVID-19) point-of-care (PoC) molecular diagnostic kit. The study used swab (NS), saliva (IS and EMS), and VTM (NOS) samples. RNA extraction was performed using a 3D printed cassette instead of a centrifuge. Reverse transcription loop-mediated isothermal amplification (RT-LAMP) assays were run in heating blocks at 65ºC for 30 min, giving pink for negative and yellow for positive results.
